# The Duty on Scientific Books and Instruments

**Published:** 1885-10

**Authors:** 


### THE DUTY ON SCIENTIFIC BOOKS AND INSTRUMENTS.

We have formerly alluded to this subject in the columns of our journal, but it is of such importance, and there is so much injustice connected with it, that it deserves not only repeated, but earnest attention.

The medical editors of the country have not taken any notice of the subject. The reason may be sought in the fact that many of the journals conducted by them are really owned by publishers who simply use the journals as a business medium for advertising their own publications, caring little for the advancement of the profession, or for the members increasing their knowledge through foreign literature.

Again, a great injustice is done those individual members of the profession who really care to elevate themselves, from the fact that the schools can obtain books and instruments free of duty, but the laity or the teachers at the schools cannot for their own use.

While at the school the poor student can have access to foreign books and the use of instruments, but when he leaves it, both are shut off from him, no matter how much he may desire to study, or to make researches, on account of the extra cost of implements for his work.

We have heretofore styled this duty on these things as an embargo on knowledge and a premium on ignorance placed by the government on the scientific profession.

Why schools should be thus favored and the really enthusiastic scientists, whose work would really be of benefit to the country, be thus hindered in their work is a problem difficult to solve. If there be any independence of thought and action in medical journalism in this country; if there be any common sense and a true appreciation of justice in the medical profession; if there be any desire to improve and advance the public weal among the editors of our daily press, this important topic will not be left any longer to the advocacy of a single medical journal in the United States.

